# Editorial: Drought Threat: Responses and Molecular-Genetic Mechanisms of Adaptation and Tolerance in Wheat

**DOI:** 10.3389/fpls.2022.960162

**Published:** 2022-06-30

**Authors:** Dev Mani Pandey, Yin-Gang Hu, Yuri Shavrukov, Narendra Kumar Gupta

**Affiliations:** ^1^Department of Bioengineering and Biotechnology, Birla Institute of Technology, Ranchi, India; ^2^State Key Laboratory of Crop Stress Biology for Arid Areas, College of Agronomy, Northwest A&F University, Xianyang, China; ^3^College of Science and Engineering (Biological Sciences), Flinders University, Adelaide, SA, Australia; ^4^Sri Karan Narendra Agriculture University, Jaipur, India

**Keywords:** wheat, drought, gene expression, growth and development, grain yield

Sessile plants must combat many challenging environments and conditions, of which drought poses one of the greatest threats. Despite noteworthy improvements in crop breeding and modern agricultural management practices, drought continues to pose the most serious challenge to agricultural production. The Research Topic (RT) presented herein aimed to address the gaps in our knowledge of how plants can effectively manage drought conditions and what elements are the most critical in this adaptation process.

Bread wheat (*Triticum aestivum* L.) is an important cereal crop grown in semi-arid and temperate regions of the world (Wan et al.). It supplies 20–30% of calories globally (Lobell and Gourdji, 2012), and is grown extensively for both for humans and livestock. Over the past two decades, global wheat yield has increased by a mere 1.0% per year (Manes et al., 2012) since it is very sensitive to adverse environmental conditions like drought, and other abiotic stresses that threaten global food security such as heat, salinity and flooding. It is expected that demand for wheat will increase by 60% by 2050, however, production may decrease by 29% as a result of climate change-imposed environmental stresses (Manickavelu et al., 2012). Without doubt, increasing drought tolerance in wheat is critical for sustainable food production and global food security (Kulkarni et al., 2017). Therefore, the development of wheat varieties with better tolerance and adaptation to drought is essential.

Many studies related to drought responses in wheat have been carried out recently describing key genes and transcription regulators involved in regulating the main morpho-physiological traits (Bi et al., 2016; Poersch-Bortolon et al., 2016; Kulkarni et al., 2017; Liu et al., 2018; El-Esawi et al., 2019; Zotova et al., 2019, 2020). Gene expression study has also been conducted in wheat grown under drought and heat stresses, indicating combined effects (Alsamman et al., 2021). Plant responses to drought are complex, where acquired changes at multiple levels combine to alter plant morphology, cell biochemistry and gene regulation. Further, efforts toward improving drought tolerance in wheat through various gene analyses have also been made (Shavrukov et al., 2016; Bi et al., 2018). Additionally, transgenic and genome editing based approaches have been employed to improve wheat resistance to drought (Yadav et al., 2015; Borisjuk et al., 2019). However, detailed studies related to wheat plant growth and development, morphology, physiology and biochemistry in response to drought stress need further attention. This includes the essential processes of carbohydrate synthesis and metabolism in grain; grain yield and quality under the stress; genomics, transcriptomics and molecular marker analyses in drought-affected wheat plants. Finally, gene identification and functional analysis of the regulation of these genes in dry environments, as well as gene editing approaches, are extremely important for the development of drought tolerant wheat plants.

The goal of the presented Research Topic was to show the current level of research and progress in the study of plant adaptation and tolerance to drought in wheat. This has encompassed research from a range of scales, from the whole plant to the molecular level, including gene network studies between tolerant and sensitive plants. A study of dynamic regulatory gene and protein networks was carried out in wheat roots under drought using a comparative transcriptomics approach (Rahimi et al.). The positive regulation of nucleoredoxin gene *TaNRX1* was found for drought tolerance in transgenic bread wheat plants (Zhang et al.). Presented results for multi-locus genome-wide association study for grain weight-related traits under rain-fed conditions in wheat can significantly improve our knowledge in this area (Gahlaut et al.). The paper published on genome-wide association study can undoubtedly aid in the identification of novel quantitative trait nucleotides for water-soluble carbohydrate accumulation in wheat plants under drought stress (Gaur et al.). The transcriptomic analysis of wheat plants revealed a hormone-mediated balance occurring during the rehydration process of plants (Liu et al.). One of the most exciting and interesting pieces of research was the association mapping study on drought tolerance in exotic Ethiopian durum wheats (Negisho et al.). In contrast, a global meta-analysis was presented on the environmental impact of drought on the yield and protein content of wheat (Wan et al.). All papers presented in the current Research Topic were based on a wide and diverse range of modern technologies, scientific approaches and research ideas aimed toward achieving a better understanding of all aspects of plant responses to drought to increase the overall tolerance of bread wheat varieties.

## Author Contributions

DP and YS prepared the manuscript. Y-GH and NG along with DP and YS edited the manuscript. All editors have read and agreed with manuscript.

## Conflict of Interest

The authors declare that the research was conducted in the absence of any commercial or financial relationships that could be construed as a potential conflict of interest.

## Publisher's Note

All claims expressed in this article are solely those of the authors and do not necessarily represent those of their affiliated organizations, or those of the publisher, the editors and the reviewers. Any product that may be evaluated in this article, or claim that may be made by its manufacturer, is not guaranteed or endorsed by the publisher.
